# Oral Glycogenic Acanthosis: A Clinicopathological Analysis of 13 Cases and Narrative Literature Review

**DOI:** 10.1007/s12105-026-01891-6

**Published:** 2026-02-19

**Authors:** Gisele N. Mainville, Safae Benali Lietefti, Antonio Nanci, Catherine Laliberté, Adel Kauzman

**Affiliations:** 1 Department of Stomatology, Faculty of Dental Medicine, Université de Montréal, Centre-ville Station, PO Box 6128, Montreal, QC H3C 3J7 Canada; 2 Department of Biochemistry and Molecular Medicine, Faculty of Medicine, Université de Montréal, Montreal, QC Canada; 3 Faculty of Dental Medicine, Université Laval, Quebec, QC Canada

**Keywords:** Glycogenic acanthosis, Extra-esophageal, Oral cavity, Oral lesions, Oral leukoplakia, Oral potentially malignant disorder

## Abstract

**Purpose:**

Glycogenic acanthosis (GA) is a benign epithelial lesion characterized by intracellular glycogen accumulation, most seen in the esophagus. In the oral cavity, it is rarely reported and clinical recognition is low.

**Methods:**

We retrospectively analyzed 13 cases of oral GA diagnosed between 2017 and 2025, assessing clinical presentation, histopathological characteristics and follow-up data.

**Results:**

Thirteen cases were identified, with a median age of 61 years (range: 34–71) and a male predominance (76.9%). Lesions were asymptomatic, thin, homogeneous, often well-demarcated milky white plaques, located on the lateral or ventral surfaces of the tongue (84.6%) or the soft palate (15.4%), all mimicking oral leukoplakia. All patients reported alcohol use; 46.2% were never smokers. Histologically, lesions showed glycogen-rich clear cells in the spinous layer of epithelium, without dysplasia. Follow-up data (available in 9 cases, 3 months to 8 years) showed that most lesions remained stable following initial diagnosis, except for one case that regressed by 80% without treatment after nearly 4 years, and another that focally progressed to mild dysplasia after 7 years in a smoking patient with HIV.

**Conclusion:**

This study presents the largest series of oral GA to date and proposes a list of distinguishing clinical and histopathological features to assist in the diagnosis of this condition. Our findings are consistent with the seven previously published reports, further supporting that oral GA is an under-recognized lesion that can clinically mimic homogenous leukoplakia. While current evidence suggests a benign long-term course, additional studies using standardized diagnostic criteria and molecular approaches are needed to better understand its pathogenesis and prognosis. Greater clinician awareness is essential to minimize misdiagnosis and ensure appropriate management.

**Supplementary Information:**

The online version contains supplementary material available at 10.1007/s12105-026-01891-6.

## Introduction

Glycogenic acanthosis (GA) is a benign mucosal lesion most commonly observed in the esophagus. It is frequently discovered incidentally during endoscopy, particularly in older adults [1]. First described in 1970 [2], GA is characterized on both endoscopy and double-contrast esophagography by numerous small, elevated white plaques that typically measure a few millimeters in diameter and may merge, creating a finely nodular or cobblestone-like mucosal appearance [3–5]. The prevalence of esophageal GA increases with age, findings reported in approximately 3.5% to 28.3% of adults, particularly in middle-aged to older populations [1, 4–6]. Histologically, lesions present acanthosis with abundant intracellular glycogen in the keratinocytes of the spinous layer [2, 5]. Although GA is well-described in the esophagus, its involvement at extraesophageal sites is rare and clinically compelling reports of GA affecting the larynx and oral cavity remain exceedingly uncommon [7–26]. Worldwide, there are fewer than twenty reported cases of GA in the oral cavity, and the first cases were reported in a 1993 abstract by Summerlin and Tomich [26].

According to the published cases, oral GA clinically mimics more common oral lesions, notably oral leukoplakia (OL), as it generally presents as a well-defined, asymptomatic white plaque. Lesions have been reported at various sites in the oral cavity, including the tongue (particularly the ventrolateral surface), the buccal mucosa, the floor of the mouth, the gingiva, and, in extremely rare instances, the lips [9–26]. While the exact etiology of oral GA remains unclear, esophageal GA has been associated with aging and gastroesophageal reflux disease (GERD) [1, 27]. GA has also been described in patients with multiple hamartoma syndrome (Cowden syndrome; CS) [28].

Histopathologically, oral GA mirrors its esophageal counterpart. It is characterized by acanthotic squamous epithelium and abundant intracytoplasmic glycogen within keratinocytes, confirmed by periodic acid-Schiff (PAS) positivity and diastase (PAS-D) sensitivity. Inflammation is typically absent, and there is no reported evidence of oral epithelial dysplasia (OED) [14].

The seemingly low prevalence of oral GA could be attributed to under-recognition and limited awareness among dental and medical professionals. The incidental identification of a white plaque, varying in size and shape, with crisply defined borders and no obvious cause, typically prompts a clinical diagnosis of OL. This is of significant importance, as OL is an oral potentially malignant disorder (OPMD), but its malignant transformation potential to oral squamous cell carcinoma (OSCC) is highly variable, with rates reported to range from 0.13% to over 40% in the literature depending on clinical subtype, anatomical site, presence and grade of epithelial dysplasia, risk factor exposure, and duration of follow-up [29–34]. Most patients presenting with OL have traditional risk factors such as long-term smoking, alcohol use, or betel quid chewing, while others may be immunocompromised or have genetic predispositions [35].

There are currently no established guidelines for the diagnosis and management of oral GA. Reported cases are asymptomatic and discovered incidentally during routine dental examination. Diagnosis relies primarily on histopathologic findings following a biopsy of the lesion. Although the condition appears to be benign, comprehensive data on treatment strategies, long-term follow-up and prognosis remains limited. Consequently, clinicians may encounter challenges in making evidence-based decisions regarding the management of this entity.

Over the past five years, thirteen cases submitted to our Montreal-based oral pathology biopsy service, clinically diagnosed as thin, homogeneous OL, were found to be histopathologically consistent with oral GA. In this retrospective study, we analyzed these cases making this the largest series of oral GA in the English literature to date. To contextualize our results and interpretations, a comprehensive literature review was performed by searching the medical databases for all published cases of oral GA. The clinical spectrum of oral GA will be discussed, along with its clinical and histopathological differential diagnosis. According to the current state of knowledge, an initial set of clinicopathological diagnostic criteria are proposed, along with recommended management strategies. Our primary goal is to increase awareness of this supposedly rare oral entity amongst clinicians and pathologists.

## Materials and Methods

This study was approved by the Ethics Committee of the Université de Montréal (2024–5750), including a waiver of informed consent.

### Patients

Thirteen patients diagnosed with oral GA at the Service de Pathologie Buccale CMB (Montreal, Quebec, Canada) between 2017 and 2025 were included in this retrospective study. All cases were retrieved through a deidentified systematic search of the pathology service database, which integrates clinical data and pathology reports. Only cases with a diagnosis of oral GA, based on clinical and histopathological features, were included. All collected data remained de-identified to ensure patient anonymity.

### Clinical Data and Statistical Analysis

Data collected focused on demographics, location in the oral cavity, clinical appearance, follow-up information, and histopathological features. In six cases, photographic documentation of the lesion was provided by the referring clinicians at the time of biopsy. The reported clinical appearance of each lesion was correlated with the histopathological findings to support a diagnosis of oral GA. All original slides (H&E, PAS, PAS-D) were reviewed by two oral pathologists (A.K. and G.M.), who reached consensus on the diagnosis and the key microscopic features. Descriptive statistics were used to summarize the collected data.

### Literature Review

A literature search was initially conducted in January 2024 using the National Library of Medicine (PubMed), with subsequent updates performed through Embase (via Ovid), to retrieve all available literature on oral GA. The final update was completed in September 2025. The search strategy used a combination of free-text keywords such as “oral” OR “extraesophageal” combined with “glycogenic acanthosis” alongside database-specific controlled vocabulary (MeSH or EMTREE). No date restrictions were applied. To ensure the inclusion of grey literature and publications not indexed in standard bibliographic databases, additional searches were conducted using Google Scholar and Lens [36]. We also hand-searched *Oral Surgery*,* Oral Medicine*,* Oral Pathology*, *and Oral Radiology *(OOOO Journal) and *Head and Neck Pathology* from 2022 to 2025, after a manual search of the OOOO Journal revealed a study that had not been previously cited or indexed. For full search strategies, see Supplementary Material.

We included case reports and abstracts that provided sufficient details and a reliable clinico-pathologic correlation for a presumptive diagnosis of oral GA, regardless of patient age or gender. Publications in languages other than English or French, or those that lacked detailed clinical or histopathological data, were excluded.

All search entries were reviewed by three independent authors (S.B.L & G.M & A.K) based on the predefined inclusion and exclusion criteria. Articles were initially screened by title and abstract, with relevant full texts subsequently reviewed. The reference lists of included articles were manually searched to identify additional eligible publications. Any discrepancies between the three reviewers were resolved through discussion and consensus. The extracted data included year of publication, patient demographics, clinical presentation, lesion characteristics, diagnostic methods, histopathological features, and any follow-up data. The analysis involved descriptive statistics and thematic synthesis of the findings.

## Results

### Case Series

Thirteen cases of oral white lesions were diagnosed as GA between 2017 and 2025. The patients’ median age at diagnosis was 61 years (range: 34–71 years). There was a clear male predominance, with 10 males (76.9%) and 3 females (23.1%), resulting in a male-to-female ratio of 3.3:1. Eleven of the thirteen cases were patients biopsied and followed by oral medicine and oral pathology (OMOP) specialists.

**Fig. 1:**
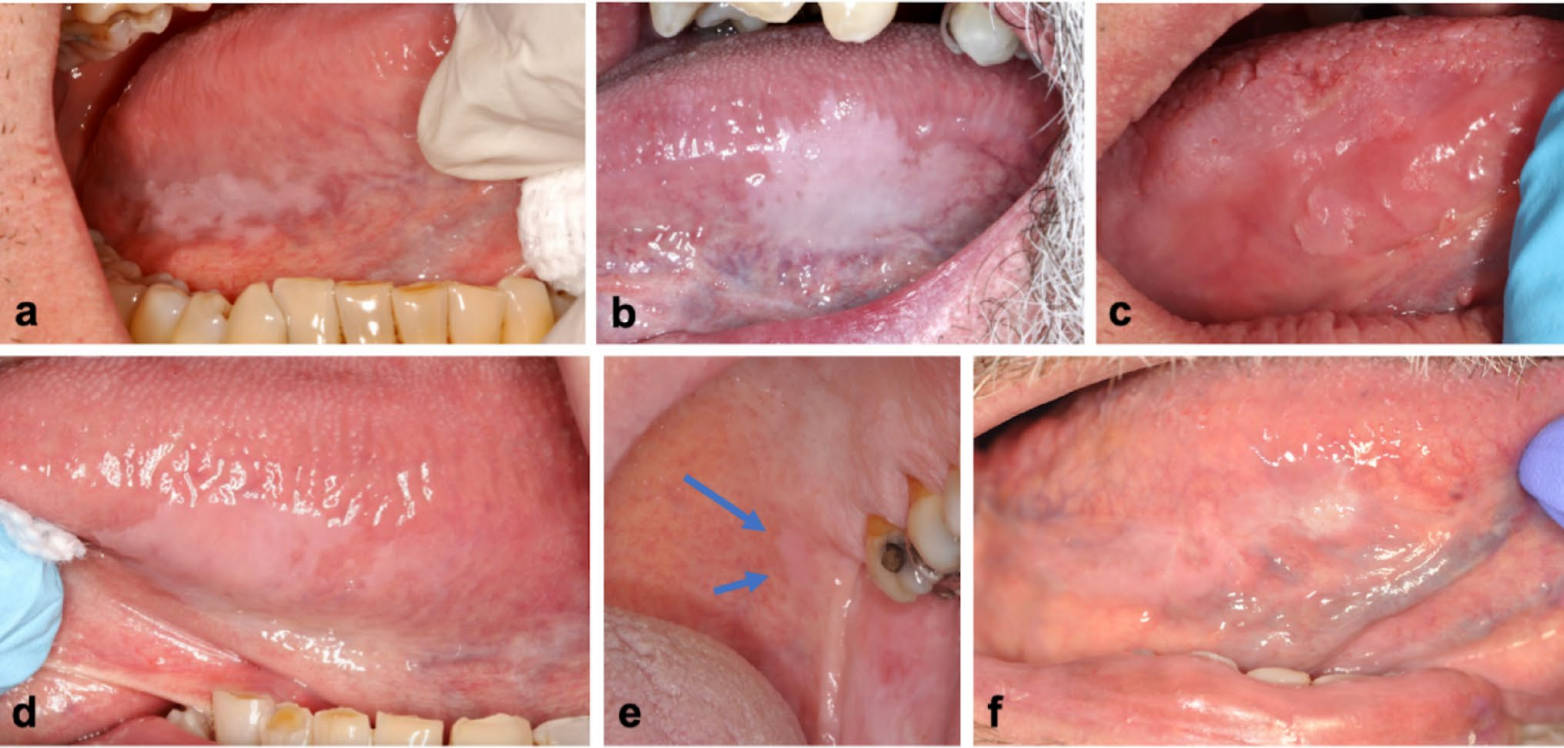
Clinical presentation of oral GA in 6 different cases. **a** Thin, homogeneous mikly white plaque on the posterior right ventrolateral surface of the tongue, with an irregular but well-defined border, measuring 25 mm × 8 mm (Case 1) **b** Smooth, thick, milky white plaque measuring 24 mm× 20 mm on the left lateral surface of the tongue (Case 7) **c** Slightly papillary pink plaque, with well-defined borders, measuring 5 mm x 4 mm, located on the right lateral surface of the tongue. The patient also has a geographic and fissured tongue (Case 10) **d** Soft, homogeneous, well-defined milky white plaques on the left ventrolateral surface of the tongue, the largest measuring 15 mm × 11 mm. A smaller second lesion is present more posteriorly (Case 13) **e** Very thin, homogeneous mikly white plaque located on the left side of the soft palate (arrows), measuring 6 mm x 6 mm (Case 12) **f** Thin white plaque on the right ventrolateral surface of the tongue, measuring 20 mm x 6 mm, with a focal thickened area at the anterior aspect. Biopsy of this area showed mild epithelial dysplasia (Case 2, at 7-year follow up)

The most frequently affected site was the lateral and ventral surfaces of the tongue (*n* = 11, 84.6%), followed by the soft palate (*n* = 2, 15.4%). Clinically, the lesions were most commonly described as asymptomatic, smooth, thin, homogeneous, milky-white unifocal plaques, typically with well-demarcated borders and no associated erythema, induration, or local irritant (Fig. 1). In all 13 cases, the referring clinician’s primary differential diagnosis was oral OL. The size of the oral lesions varied, ranging from 5 mm to 25 mm in widest dimension.

For all patients, a history of alcohol use was provided by the clinician submitting the biopsy. Alcohol consumption was classified as rare (very infrequent use, a few times per year; *n* = 1, 7.7%), occasional (up to 1–2 drinks per week or a few times per month; *n* = 7, 53.8%), moderate (regular intake of about 2–3 drinks per week on a long-term basis; *n* = 1, 7.7%), or frequent (high or daily alcohol consumption, ≥ 7 drinks per week or several drinks per day; *n* = 4, 30.8%). For two cases with frequent alcohol consumption, the use of alcohol-containing mouthwash was also reported. Three patients were current smokers, with tobacco use ranging from 25 to 80 pack years. Six patients were never smokers (46.2%), and four were ex-smokers, having quit between 4 and 30 years prior, and having smoked between 3 and 25 pack years. Two patients had a known diagnosis of GERD, another had suspected but undiagnosed GERD, and none had a diagnosis of CS. Other comorbidities included hypertension (*n* = 2, 15.4%), dyslipidemia (*n* = 2, 15.4%), depression (*n* = 2, 15.4%) and a history of cancer (laryngeal, prostate, and skin) (*n* = 2, 15.4%). Ethnicity data was not provided by the submitting clinicians.

**Fig. 2:**
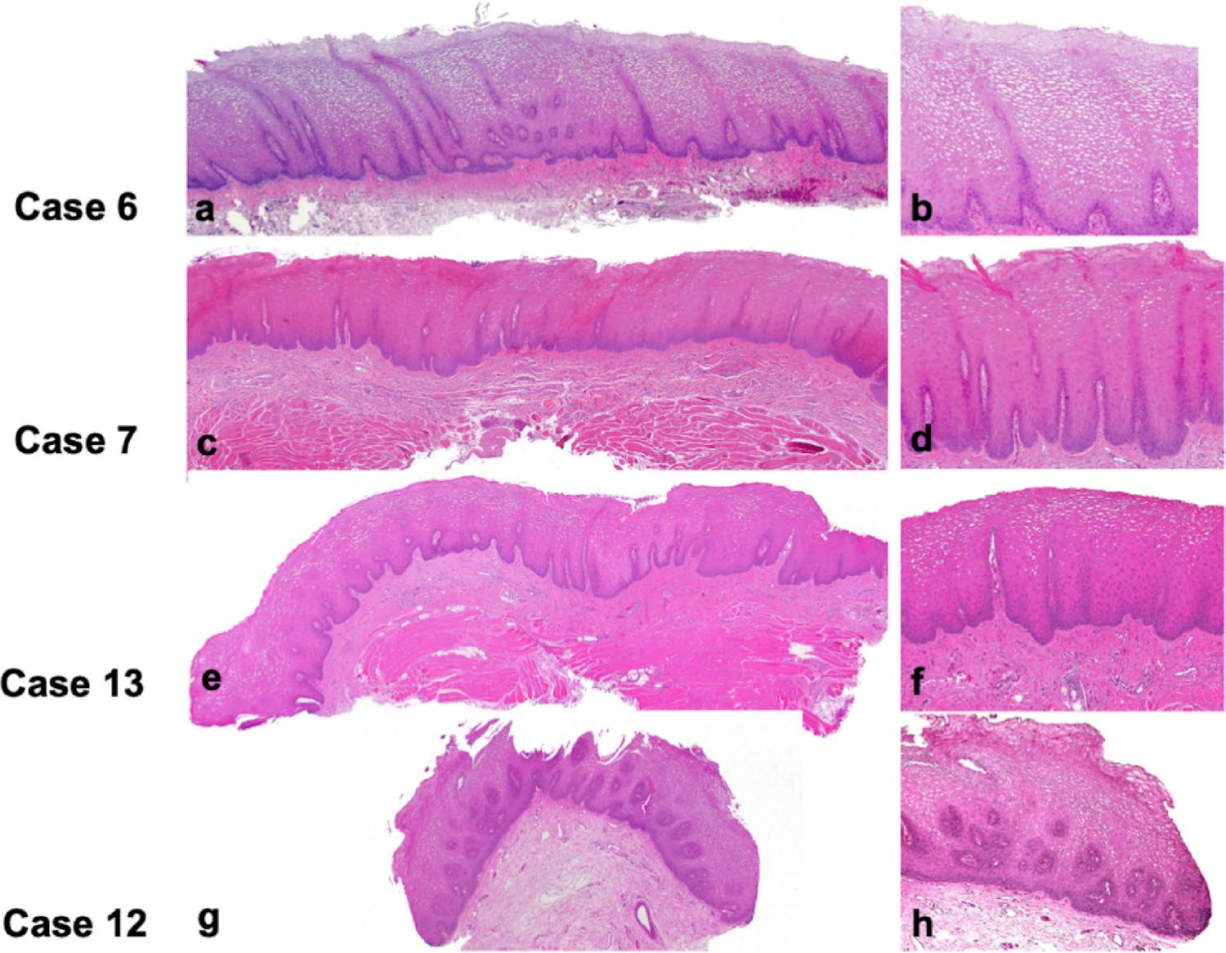
Histopathological features 4 representative cases of oral GA. **a**,** c**,** e**,** g** show parakeratinized, acanthotic stratified squamous epitheliumwith no signs of epithelial dysplasia (H&E, original magnification 2.5 x) **b**,** d**,** f**,** h** show keratinocytes with clear, glycogen-rich cytoplasm in the spinous layer. Aside from mild basal hyperplasia, the epithelial maturation pattern is normal, without an inflammatory host response (H&E, original magnification 10 x)

**Fig. 3:**
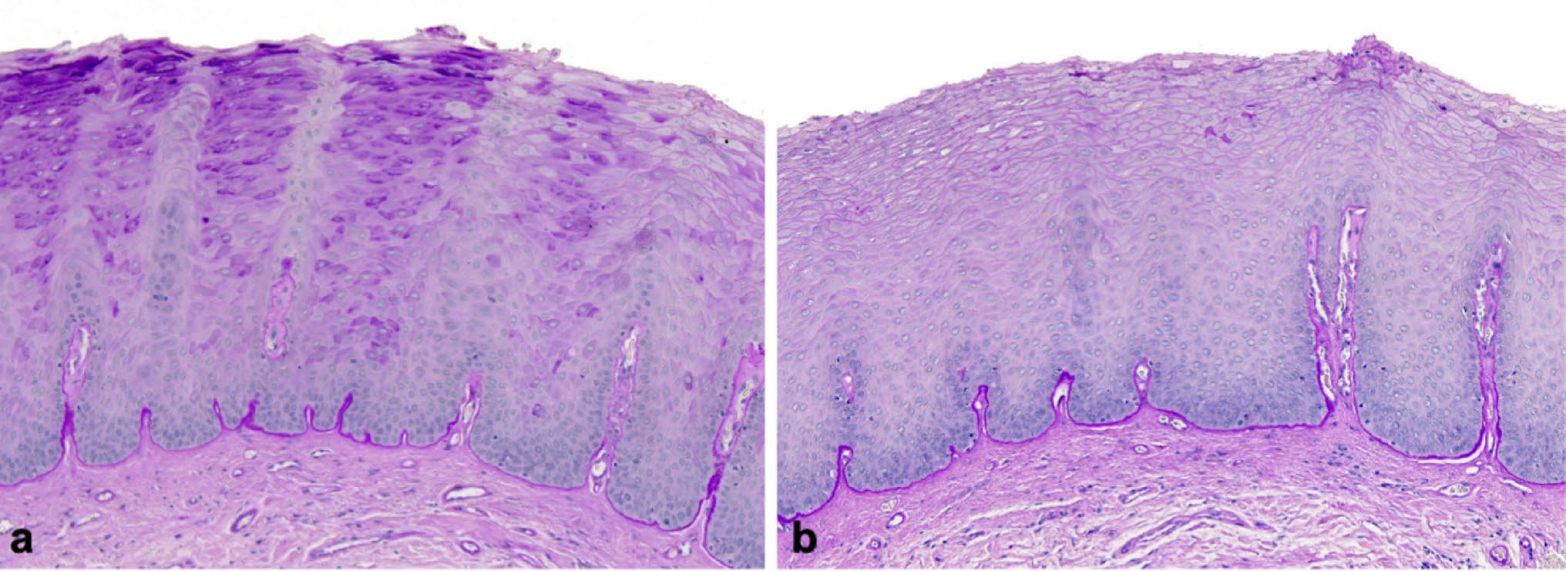
Representative example of intraepithelial glycogen in oral GA (case 7) **a** Intense magenta cytoplasmic staining of suprabasal keratinocytes, and **b** complete disappearance following diastase digestion, confirming the glycogen content (PAS, original magnification 10 x)

Microscopically, all cases exhibited variably hyperparakeratinized and acanthotic stratified squamous epithelium (Fig. 2). Short parakeratin chevrons were occasionally present (Figs. 2a and d and 5a and b). The hallmark feature was the presence of abundant clear cells in the spinous layer. In all 13 cases, these cells were PAS-positive and diastase-sensitive, confirming their glycogen content (Fig. 3). Importantly, there was no evidence of OED nor malignancy at the time of the initial histopathologic analysis.

**Fig. 4:**
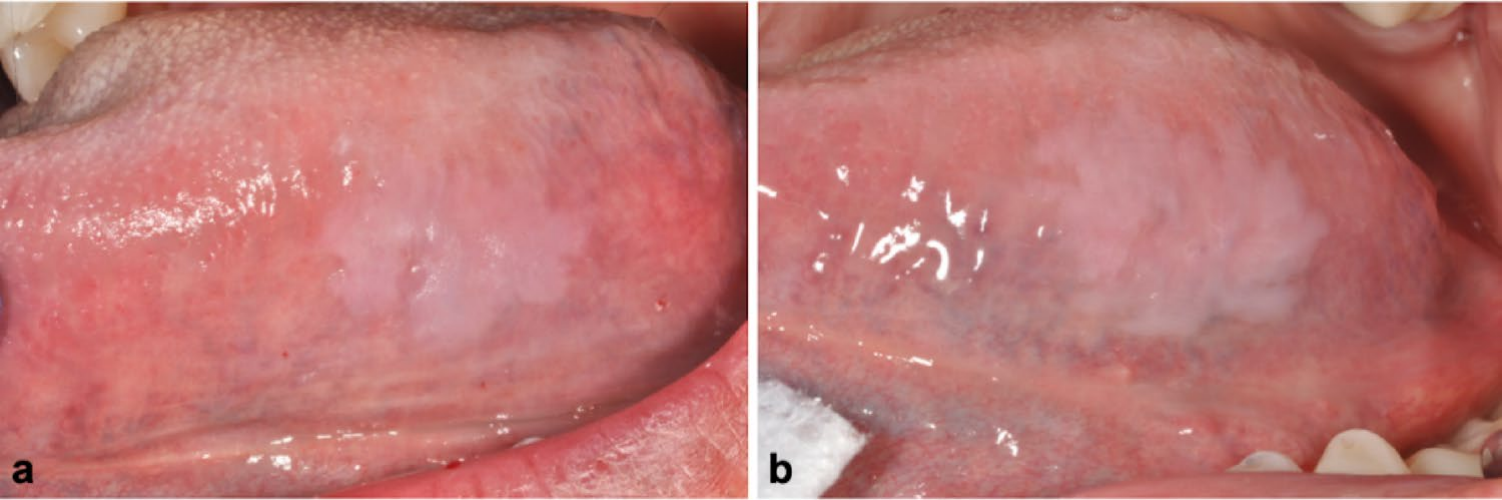
Slight enlargement of an oral GA lesion after one year (case 8). **a** Initial presentation showing a thin, smooth, well-demarcated, milky-white plaque on the posterior left lateral surface of the tongue, measuring 15 mm x 10 mm **b** One year later, the lesion showed slight thickening, as well as an increase in size (20 mm x 12 mm). It remained stable for the next 32 months and continues to be followed

**Fig. 5:**
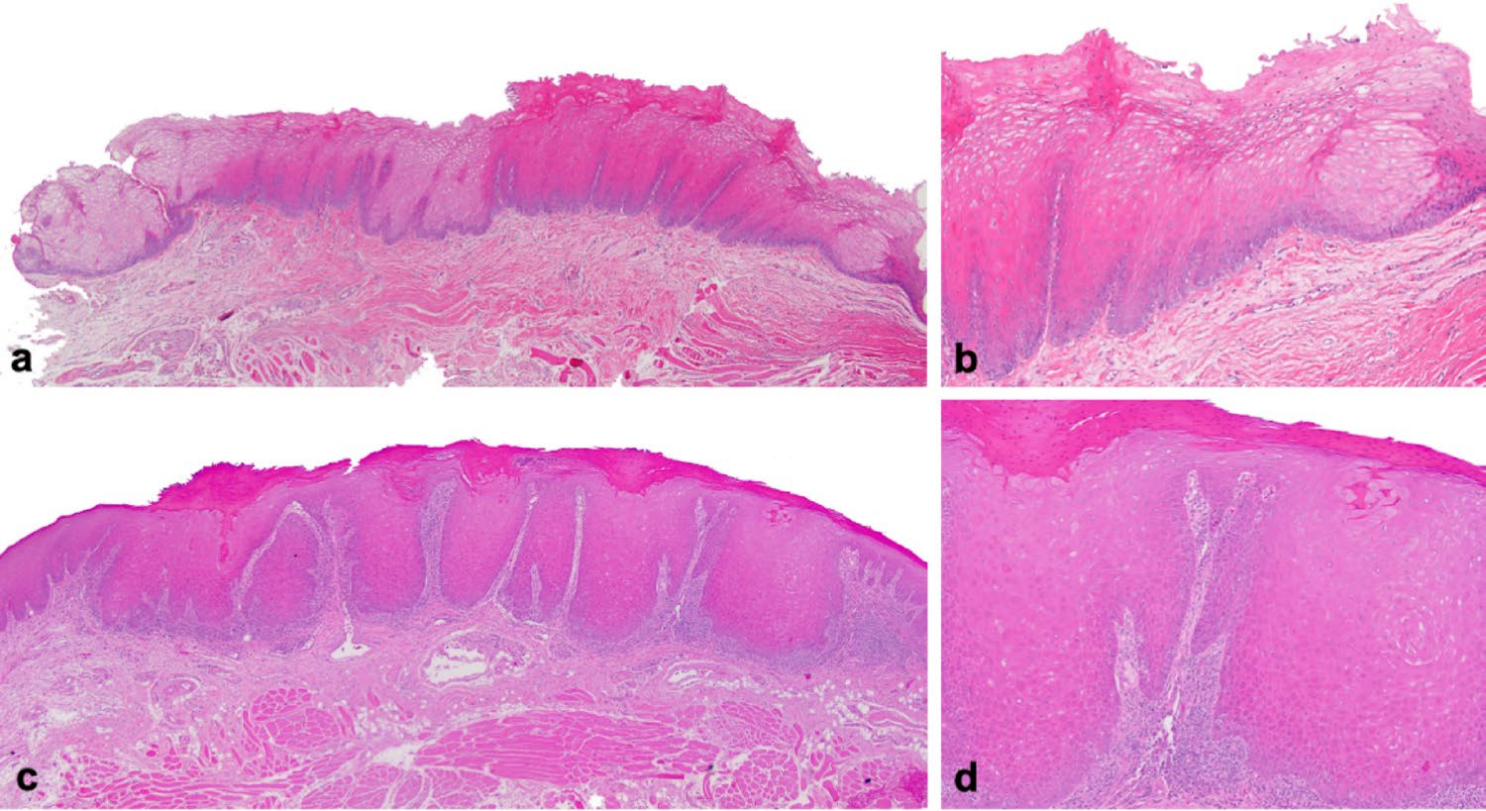
Histopathological features of oral GA showing focal progression to mild dysplasia after 7 years of follow-up in an HIV-positive cigarette-smoking patient (case 2). **a** Initial incisional biopsy specimen from the lateral surface of the tongue showing hyperparakeratinized and acanthotic stratified squamous epithelium forming narrow rete ridges and parakeratin chevrons. There is no epithelial dysplasia or inflammatory host response (H&E, original magnification 2.5 x) **b** Higher magnification showing cytoplasmic vacuolization of keratinocytes in the spinous layer. Basal cell hyperplasia is observed, but the maturation pattern is otherwise normal (H&E, original magnification 10 x) **c** Second incisional biopsy taken 7 years later (lesion seen in Fig. 1f) demonstrating mild epithelial dysplasia. (H&E, original magnification 2.5 x) **d** Dyskeratosis, bulbous rete ridges and a lymphocytic host response are visible. PAS staining ruled out superficial candidal infection. (H&E, original magnification 10 x)

**Fig. 6:**
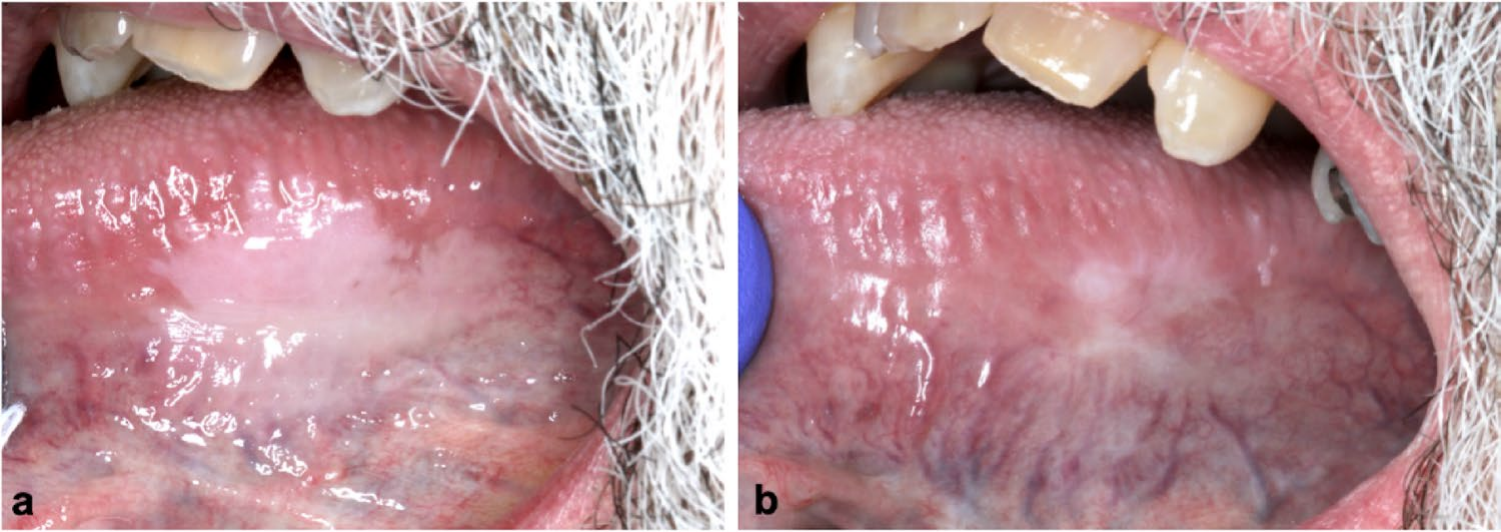
Near complete spontaneous regression of an oral GA lesion after 45 months (case 7). **a** 5 months after the initial presentation (lesion seen in Fig. 1b) and incisional biopsy, the lesion remained unchanged **b** At a routine follow-up appointment 45 months after the initial presentation, the lesion had almost completely regressed without treatment. The patient reported occasional GERD which was not treated

All patients were offered long-term clinical monitoring and, when needed, counseling regarding tobacco and alcohol cessation. Follow-up data were available for 9 patients (69.2%), with monitoring periods ranging from 3 months to 8 years. The majority (*n* = 6, 66.7%) of the lesions were stable over time without progression or recurrence after excisional biopsy (when performed). One case showed a minor increase in lesion size (from 15 mm x 10 mm to 20 mm x 12 mm) after a follow-up period of 12 months, then remained stable for the next 32 months (Fig. 4). Another case demonstrated focal progression to mild dysplasia over a 7-year period; this patient was a long-time smoker and was HIV positive (Figs. 1h and 5). Finally, a third patient with occasional, untreated GERD had a 80% decrease in their lesion 45 months following incisional biopsy (Figs. 1b and 6). A detailed summary of the included cases is presented in Table 1.

**Table 1:** Clinicopathologic features of all reported cases of oral GA, including our 13 cases

Author (year), Country	Sex	Age	Medical history	Alcohol*	Tobacco	Oral site	Lesion history	Size (mm)	Clinical description	Clinical diagnosis	Histopathologic features	Treatment	Follow-up (months); outcome
Mainville et al. (2026), Canada Case 1	44	M	Hypertension	Occasional	Ex-smoker, quit 4 years prior, 9 pack years	Posterior ventrolateral tongue	Asymptomatic, noted by dentist 4 years prior, stable	25 × 8	Thin homogenous milky white plaque with irregular but well-defined borders. No local irritant.	OL	Parakeratosis with parakeratin chevrons, acanthosis with basal hyperplasia, clear glycogen-rich keratinocytes in the spinous layer confirmed with PAS/PAS-D, mild chronic inflammation	Incisional biopsy, follow-up	72; lesion stable
Case 2	67	M	HIV+, Focal epithelial hyperplasia, dyslipidemia, COPD	Occasional	Current smoker, 25 pack years	Posterior ventrolateral tongue	Asymptomatic, unknown duration	20 × 3	Partially-well-defined thin white homogenous plaque	OL	Parakeratosis with parakeratin chevrons, acanthosis with basal hyperplasia, clear glycogen-rich keratinocytes in the spinous layer confirmed with PAS/PAS-D. No fungal infection.	2 incisional biopsies 7 years apart, follow-up	96; lesion focally progressed to mild dysplasia after 7 years, then remained stable
Case 3	67	M	NR	Occasional	Never	Lateral tongue	NR	15 × 10	White plaque with irregular borders	OL	Parakeratosis, acanthosis with clear glycogen-rich keratinocytes in the spinous layer confirmed with PAS/PAS-D, mild chronic inflammation	Incisional biopsy	Lost to follow-up
Case 4	54	F	Hypertension	Moderate	Never	Posterior ventrolateral tongue	Asymptomatic, unknown duration	10 × 6	Pale homogenous white plaque with partially well-defined borders. No local irritant	OL	Hyperparakeratosis, acanthosis with basal hyperplasia, clear glycogen-rich keratinocytes in the spinous layer confirmed with PAS/PAS-D, non-inflamed connective tissue	Excisional biopsy, follow-up	3; no recurrence, then lost to follow-up
Case 5	71	F	GERD, Hyperthyroidism, Asthma	Rare	Current smoker, 51 pack years	Soft palate at junction with maxillary tuberosity, bilaterally	Asymptomatic, unknown duration	10 × 5 15 × 5	2 well-demarcated thin white plaques	OL	Hyperparakeratosis with parakeratin chevrons, acanthosis with basal hyperplasia, clear glycogen-rich keratinocytes in the spinous layer confirmed with PAS/PAS-D, mild chronic inflammation	Incisional biopsy	Lost to follow-up
Case 6	70	M	Laryngeal cancer, prostate cancer	Occasional	Never	Lateral tongue	Asymptomatic, unknown duration	8 × 8	Round well-demarcated white plaque with subtle texture	OL or OHL	Hyperparakeratosis, acanthosis, clear glycogen-rich keratinocytes in the spinous layer confirmed with PAS/PAS-D. non-inflamed connective tissue	Excisional biopsy, follow-up	34; no recurrence
Case 7	62	M	Occasional GERD (untreated), depression	Frequent	Never	Lateral tongue	Asymptomatic, present less than 6 months	24 × 20	Soft, smooth, homogenous milky white plaque	OL or GA	Hyperparakeratosis, acanthosis with basal hyperplasia, clear glycogen-rich keratinocytes in the spinous layer confirmed with PAS/PAS-D, candidiasis	Incisional biopsy, follow-up	45; lesion regressed by 80% without intervention
Case 8	53	M	Depression	Frequent. Also, occasional use of ETOH-containing mouthwash.	Ex-smoker, quit 10 years prior, 25 pack years	Lateral tongue	Asymptomatic, present at least 6 months	15 × 10	Thin, smooth well-demarcated milky-white plaque	OL	Hyperparakeratosis with parakeratin chevrons, acanthosis with basal hyperplasia, clear glycogen-rich keratinocytes in the spinous layer confirmed with PAS/PAS-D, non-inflamed connective tissue	Incisional biopsy, follow-up	44; lesion enlarged slightly to 20 mm x 12 mm after one year, then remained stable
Case 9	55	M	Dyslipidemia, Skin cancer	Frequent. Also, daily use of ETOH-containing mouthwash x 30 years	Current smoker, 80 pack years	Ventral tongue	Asymptomatic, noted by dentist a few weeks prior	8 × 3	Thin homogenous well-demarcated white plaque	OL	Hyperparakeratosis, acanthosis with basal hyperplasia, clear glycogen-rich keratinocytes in the spinous layer confirmed with PAS/PAS-D, mild chronic inflammation	Excisional biopsy	Lost to follow-up
Case 10	69	M	Arthrosis	Occasional	Never	Lateral tongue	Asymptomatic, noted by dentist 4 years prior, stable	5 × 4	Slightly papillary pink plaque with well-defined borders	OL, squamous papilloma, or VX	Parakeratosis, acanthosis with basal hyperplasia, clear glycogen-rich keratinocytes in the spinous layer confirmed with PAS/PAS-D, non-inflamed connective tissue	Excisional biopsy, follow-up	6; no recurrence
Case 11	34	F	Rectitis	Occasional	Never	Ventrolateral tongue	Asymptomatic, unknown duration	20 × 10	Homogenous well-defined thin white plaque	OL	Hyperparakeratosis, acanthosis with basal hyperplasia, clear glycogen-rich keratinocytes in the spinous layer confirmed with PAS/PAS-D, mild chronic inflammation	Incisional biopsy, follow-up	3; lesion stable
Case 12	61	M	Dyslipidemia	Frequent	Ex-smoker, quit 30 years prior, 3 pack years	Lateral soft palate	NR	6 × 6	Very thin homogenous milky white plaque	OL	Parakeratosis, acanthosis with basal hyperplasia, clear glycogen-rich keratinocytes in the spinous layer confirmed with PAS/PAS-D, non-inflamed connective tissue	Incisional biopsy	Lost to follow-up
Case 13	47	M	Asthma, glaucoma, migraines, necrotizing fasciitis (forearm), possible GERD	Occasional	Ex-smoker, quit 20 years prior	Ventrolateral tongue	Asymptomatic, present at least 3 months, noted by dentist, dental erosion	15 × 11, with smaller second lesion posteriorly	Thin homogenous, well-defined milky white plaques	OL	Parakeratosis, acanthosis with basal hyperplasia, clear glycogen-rich keratinocytes in the spinous layer confirmed with PAS/PAS-D, non-inflamed connective tissue	Incisional biopsy, follow-up, PPI recommended	8; lesion stable
Mumtaz et al. (2024), UK	68	M	NR	NR	NR	Lateral tongue	Asymptomatic	NR	Smooth, raised white plaque	NR	Hyperplastic squamous mucosa, prominent acanthosis, PAS+ glycogen in enlarged keratinocytes	Biopsy	NR
Gotur et al. (2024), India	42	M	None	NR	Gutka chewer for 8–10 years	Lateral tongue, extending to dorsal and ventral surfaces	Asymptomatic, present 6 years, slowly enlarging	40 × 60	Large, opaque white, non-scrapable smooth lesion with spongy texture, well-demarcated, no local irritant	OL	Hyperplastic epithelium, basilar hyperplasia, enlarged clear to pale keratinocytes in the suprabasilar layers, glycogen confirmed with PAS/PAS-D, no evidence of dysplasia, non-inflamed connective tissue	Incisional biopsy, follow-up	6; lesion stable
Mahmood et al. (2019), UK	78	M	Good health	Mild	Ex-smoker, quit 40 years prior	Ventral tongue	Asymptomatic, present 13 years	10 × 30	White lesion	OL	Acanthosis, glycogen-rich keratinocytes, no evidence of dysplasia	Incisional biopsies	NR
Schultz et al. (2018), Brazil	56	M	Non-contributory	None	Never	Posterior lateral tongue	Asymptomatic. Similar lesion excised 4 years prior in the same location (no dysplasia).	30 (however, lesion appears smaller in the clinical photo)	Well-defined white plaque with fluffy surface, no local irritant	OL	Parakeratosis, hyperplastic squamous epithelium, prominent acanthosis, abundant intracytoplasmic glycogen confirmed with PAS/PAS-D, no evidence of dysplasia	Excisional biopsy	16; no recurrence. Endoscopy excluded esophageal GA.
Akakhori et al. (2017), Japan	72	F	NR	NR	NR	Floor of mouth	White lesions noticed 10 days prior	NR	Plaque-like thickenings on the left side	OL	Thickened squamous epithelium with clear, enlarged keratinocytes, glycogen confirmed with PAS/PAS-D	Biopsy	NR
Fernandes et al. (2017), Brazil	56	M	NR	NR	NR	Lateral tongue	Asymptomatic white plaque that had been removed 4 years prior	30	Well-defined white plaque with verrucous surface	NR	Hyperplastic squamous epithelium with intracytoplasmic glycogen	Biopsy	NR Endoscopy excluded esophageal GA
Montebugnoli et al. (2010), Italy	72	M	Good health	NR	Never	Ventrolateral tongue	Present 5 years, biopsied initially, slowly-enlarging since then, prompting re-biopsy	40	Milky white, well-defined, plaque-like thickening, no local irritant	OL	Parakeratosis, acanthosis with basal hyperplasia, clear glycogen-rich keratinocytes in the prickle layer confirmed with PAS/PAS-D. no evidence of dysplasia, inflammation or fungal structures. HPV excluded by IHC.	Twice biopsied, 5 years apart.	NR

*Alcohol use : Rare: Very infrequent alcohol use (a few times per year), Occasional : Up to 1–2 drinks per week, or a few times per month, Moderate : Regular intake of about 2–3 drinks per week on a long-term basis, Frequent : High or daily alcohol consumption: ≥7 drinks per week, or several drinks per day

GA, glycogenic acanthosis; NR, not reported; OL, oral leukoplakia; M, male; F, female; PAS, Periodic acid-Schiff; PAS-D, PAS with diastase; IHC, immunohistochemistry; COPD, chronic obstructive pulmonary disease; ETOH, alcohol; GERD, gastroesophageal reflux disease; HPV, human papillomavirus; HIV, human immunodeficiency virus; PPI, proton pump inhibitor; VX, verruciform xanthoma

### Literature Review

A total of 18 publications reporting 19 cases of oral GA were identified [9–26]. Following title and abstract screening, all articles underwent full-text and methods review. Of these, 12 cases were excluded based on our inclusion and exclusion criteria [9–12, 15–17, 21–23, 25, 26]. One abstract included two cases; one was included, while the other was excluded [9]. Seven cases were included in the final analysis [9, 13, 14, 18–20, 24], comprising 3 case reports [14, 19, 24] and 4 conference abstracts [9, 13, 18, 20], published between 2010 and 2024. Publications originated from five different countries: Italy, Japan, Brazil, the United Kingdom, and India.

Our review of the seven retained reports of oral GA revealed that the condition predominantly affects males (85.7%, *n* = 6) [13, 14, 18–20, 24], with only one case reported in a female [9]. The median age of affected patients was 68 years, ranging from 42 to 78 years. Four cases involved patients in good health with no contributive medical history, whereas in three cases, this information was not reported. One had a history of gutka chewing [14], and another reported mild alcohol consumption and a history of smoking, having quit 40 years earlier [18]. Based on the information available in the reported cases, none of the patients were current smokers.

Clinically, lesions were consistently described as white, asymptomatic plaques. Most lesions had a uniformly smooth surface; however, two cases were described as having either a “fluffy” [24] or “verrucous” [13] surface texture. A clinical working diagnosis of OL was suggested in five cases. All cases involved either the latero-ventral surface of the tongue (71.4%, *n* = 5) or the floor of mouth (14.2%, *n* = 1) (10). One case involved both sites (14.2%, *n* = 1) [13]. Lesion size was documented in five of the seven cases, with a median dimension of 30 mm (range: 10 –60 mm) [13, 14, 18, 19, 24]. Two cases did not specify the lesion size [9, 20].

Clinical photographs were available in three case reports [14, 19, 24], and histological images were provided for the same three. Histopathological features consistently included typical characteristics of GA: acanthotic squamous epithelium, a spinous layer composed of enlarged keratinocytes with clear, glycogen-rich cytoplasm. PAS/PAS-D staining was reported in five cases [9, 14, 19, 20, 24] but was not mentioned in the other two conference abstracts. In these latter two cases [13, 18], we assumed that PAS staining was done based on the histopathological diagnostic criteria of GA that the authors provided. No dysplasia was reported in any of the cases.

Lesions were typically long-standing, with a median duration of approximately 4.5 years, ranging from 10 days to 13 years [9, 13, 14, 18, 19, 24]. Two patients reported previous excisions of similar lesions at the same site, both occurring about four years earlier, with no histological evidence of dysplasia in the earlier specimens [13, 24]. In one case, the lesion had been noticed 10 days prior to consultation [9], and in another, the duration was not documented [20]. Follow-up data were available for two cases, one showed no recurrence 16 months after excision [18], while the other remained sTable 6 months following an incisional biopsy [14]. Montebugnoli et al. [19] and Gotur et al. [14] both reported slow enlargement of a large plaque involving the ventrolateral surface of the tongue over 5 to 6 year periods, respectively, prompting biopsy. Among the seven reported cases of oral GA, two patients underwent upper endoscopic examination, with no esophageal GA observed in either case [13, 24]. A detailed summary of the included cases is presented in Table 1.

## Discussion

The present study represents the largest series of oral GA in the English literature to date. The seemingly rare occurrence of oral GA is underscored by the limited number of cases identified for inclusion in our analysis, despite an extensive literature search. As a result, understanding the true prevalence and clinical spectrum of oral GA remains a challenge. The scarcity of documented cases also contributes to a lack of widespread clinical awareness, resulting in diagnostic uncertainty among oral healthcare providers.

Based on our clinical and histopathological findings, together with the cases of oral GA previously published the literature, three hypothetical clinical scenarios of oral GA can be described, although data remain limited:

1. *Plaque-like GA* is the most frequently reported form and usually appears as a solitary, well-defined milky-white plaque, most commonly on the lateral or ventral tongue, which can clinically mimic OL.
2. *Syndromic GA* is typically associated with CS and presents with papular, widespread lesions that may involve the oral cavity, esophagus, or larynx. No cases of syndromic GA were identified in our case series, and the only case published in the literature [21] was not retained in our literature review.
3. *Nodular GA*, which remains debated, is characterized by one or a few nodules or tumor-like masses of acanthotic, glycogen-rich epithelium, occasionally appearing on the lips or gingiva. This variant may correspond to what Summerlin and Tomich described as “clear cell acanthoma” in a 1993 abstract [26]; however, these cases appear morphologically distinct and likely do not fall under the clinical spectrum of oral GA. For this reason, examples of nodular GA were not included in our literature review.

In our series, most patients were middle-aged to elderly adults, with a male predominance. Seven patients were current or ex-smokers, while six had no history of smoking. All reported some degree of alcohol consumption, ranging from occasional to frequent. The clinicopathologic features observed in our series are consistent with those reported in earlier studies [14, 19, 24]. Unfortunately, smoking and alcohol consumption habits are inconsistently documented in the literature, with only one published case reporting alcohol use [18]. This lack of uniformity limits the assessment of potential lifestyle-related etiologic factors. An age-related epithelial process in predisposed individuals remains a plausible mechanism.

Oral GA primarily involves the mobile non-keratinized mucosa of the tongue, especially its lateral and ventral surfaces [13, 14, 18–20, 24], the floor of the mouth [9, 18] or the soft palate. Clinically, these lesions are asymptomatic, typically well-demarcated, unifocal white plaques with a smooth, milky-white surface. Two cases exhibited a slightly verrucous or fluffy texture [13, 24]. All lesions raised suspicion for OL, prompting biopsy for definitive diagnosis. A history of recurrence following complete excision has been documented [13, 24], as well as slow lesional enlargement over multiple years [14, 19].

Histopathologically, oral GA is characterized by parakeratotic and acanthotic squamous epithelium with superficial keratinocytes showing glycogen-rich, clear cytoplasm. This feature is confirmed by PAS positivity and diastase sensitivity [14, 19, 24]. No epithelial dysplasia was observed in any of the cases. However, the presence of hyperparakeratosis, acanthosis and clear cells can mimic several benign conditions, including linea alba, morsicatio buccarum, leukoedema, reactive (frictional) keratosis, tobacco pouch keratosis and oral hairy leucoplakia. This emphasizes the importance of a thorough clinicopathological correlation. In fact, several cases initially reported as oral GA were excluded from our literature review, as their clinical and histopathological descriptions were far more consistent with other oral lesions [10, 16, 23]. Because oral mucosa exposed to chronic trauma may demonstrate hyperkeratosis, acanthosis, intracellular oedema and basal cell hyperplasia [37, 38], it may be difficult to distinguish oral GA from reactive white lesions. However, clinically, oral GA presents well-defined borders - a helpful feature which distinguishes it from frictional or traumatic hyperkeratosis that will show diffuse, blending borders.

The histological consistency of GA across oral [14, 19, 24], esophageal [1, 27], and laryngeal [7, 8] sites suggests a shared pathophysiologic mechanism, possibly degenerative or age-related in nature. The epithelial similarities across these anatomic sites imply that glycogen accumulation may represent a non-specific epithelial response to localized injury or irritation, though the exact triggers remain unknown [7, 24]. Nevertheless, it is clear that the lesion is not associated with systemic abnormalities in glucose metabolism, such as diabetes [39].

**Fig. 7:**
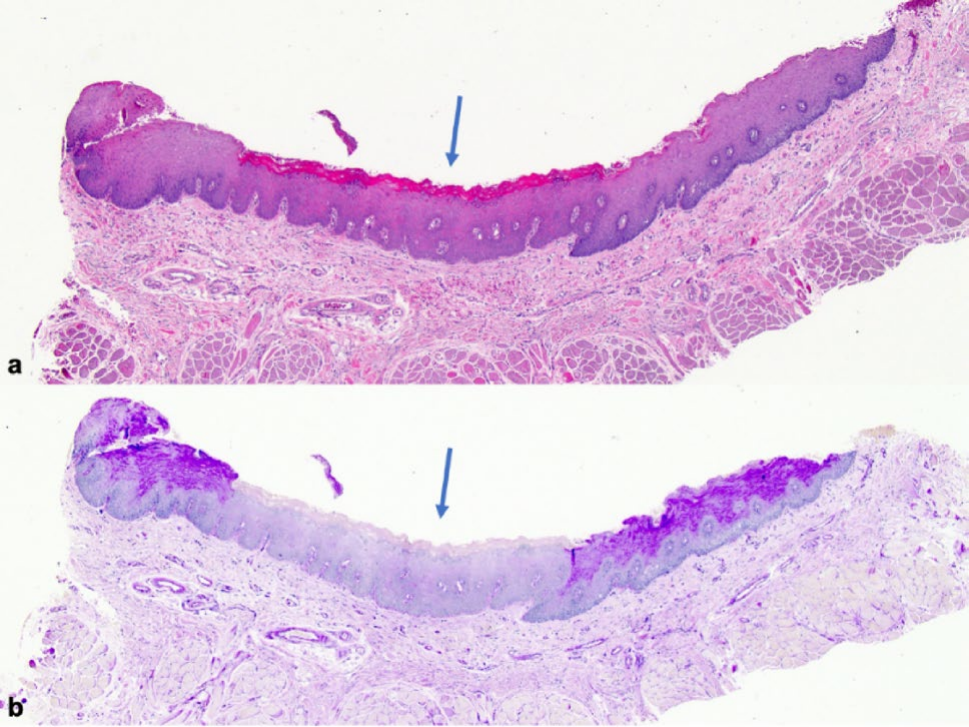
Leukoplakia of the lateral surface of the tongue demonstrating mild epithelial dysplasia. **a** A central, well-demarcated region of mild epithelial dysplasia (arrow) is bordered on each side by normal epithelium (H&E, original magnification 2.5 x) **b** The area of mild epithelial dysplasia shows no intracytoplasmic glycogen within the upper spinous layer (arrow), as compared with the normal adjacent epithelium. (PAS without diastase, original magnification 2.5 x)

The localized, well-demarcated accumulation of glycogen in a specific area of the oral mucosa is intriguing. In normal stratified squamous epithelium, glycogen is usually absent in basal cells but becomes prominent in the upper spinous layers [40], likely acting as a local energy reserve as cells migrate away from the vascular supply. Glycogen content typically declines in the superficial keratinocytes, suggesting involvement in the keratinization process [41–44]. Interestingly, while glycogen is abundant in healthy oral mucosa, its levels have been reported to decrease in pathological conditions such as OED (Fig. 7) and squamous cell carcinoma [45, 46]. The mechanism behind this reduction is unclear but may reflect increased metabolic demands or decreased glycogen synthesis [47].

One of the most significant histopathological diagnostic challenges lies in distinguishing oral GA from OL demonstrating mild OED lacking cytologic atypia, also known as keratosis of unknown significance, differentiated dysplasia or architectural dysplasia. Both conditions present clinically as well-defined white plaques, but their histological profiles differ. Typically, differentiated dysplasia displays hyperorthokeratosis and epithelial atrophy, often with abrupt transitions between normal and altered epithelium, and skip lesions [38] (Fig. 7). Although cytologic atypia may be minimal or absent, abnormal keratin production and architectural disruption in these lesions can suffice for a diagnosis of mild OED, especially under the 2022 WHO diagnostic criteria of OED [48].

In contrast, GA shows a parakeratinized, acanthotic epithelium, occasionally with short parakeratin chevrons, basilar hyperplasia, and lacks dyskeratosis, abnormal variation in nuclear and cellular size and shape and abnormal mitoses. The hallmark is the presence of glycogen-rich keratinocytes in the spinous layer, which may account for the lesion’s smooth, often milky-white appearance. Importantly, GA lacks both cytologic atypia and architectural features of dysplasia. However, histologic overlap between oral GA and mild OED can occur, particularly in borderline cases, making accurate diagnosis challenging and subjective.

In order to aid clinicians and pathologists in recognizing oral GA, we propose a list of essential clinical and histopathological diagnostic criteria (Table 2).

**Table 2:** Proposed clinical and histopathological diagnostic criteria for oral GA

Clinical criteria	Smooth, homogenous (often milky-white), keratotic plaque with (at least partially) well-defined borders, situated on mobile non-keratinized oral mucosa.
	Absence of local irritants (e.g. a sharp tooth, morsicatio, smokeless tobacco).
	Present for at least 2 weeks.
Histopathological criteria	Parakeratinized, acanthotic stratified squamous epithelium with abundant glycogen-rich keratinocytes in the spinous layer, made evident by PAS/PAS-D.
	Absence of architectural and cytologic features of OED.

Three cases in our series may provide insight into potential associated clinical factors. The first cases were two patients (cases 5 and 7) with a reported history of GERD, which could cause chronic mucosal irritation and potentially contribute to oral GA development. Interestingly, in case 7, a man in his 60s with occasional GERD, the large GA lesion involving the lateral surface of his tongue spontaneously regressed by approximately 80% over a 45-month period following incisional biopsy, without intervention or anti-acid treatment (Figs. 1b and 6). It is possible that other patients in our cohort also had GERD, but this information was not provided by the clinician. Patients also could have had silent GERD, or may not have realized they had symptoms of GERD. Although the literature describes an association between esophageal GA and GERD, in our literature review, it was interesting to note that two patients with oral GA who underwent upper endoscopy did not show signs of esophageal GA [13, 24].

The other noteworthy case from our series is a cigarette-smoking HIV-positive patient (case 2) who developed mild epithelial dysplasia adjacent to a lateral tongue GA after 7 years of follow-up (Figs. 1f and 5). This patient’s initial biopsy was diagnosed in 2017 as GA based on the absence of architectural and cytologic signs of OED, the presence of acanthosis and PAS-positive intracytoplasmic glycogen within the vacuolated keratinocytes of the spinous layer. The diagnosis was re-confirmed at the time of the study. Even with the newer 2022 WHO diagnostic criteria for OED [48] in mind, the initial biopsy did not meet histopathological criteria for OED. In this case, the development of mild dysplasia at the anterior end of the patient’s white plaque 7 years later could have represented concomitant development of OED adjacent to an area of oral GA. The patient did have risk factors for oral cancer. Alternatively, it could also indicate that oral GA can progress to OED in some cases. A previous study reported that HIV infection produces morphometric changes in oral epithelia, such as epithelial thickening, increased cell size, and glycogenic acanthosis [49]. These HIV-related modifications may weaken epithelial barrier function and cellular regulation, possibly facilitating dysplastic changes under chronic immunosuppressive conditions. Although a coincidental coexistence cannot be excluded, this finding suggests that systemic immunosuppression could influence the local epithelial environment and potentially modulate the biological behavior of GA.

At present, there is no compelling evidence that oral GA has a potential for malignant transformation. Like its esophageal counterpart, it seems to represent a benign condition. Nevertheless, genetic or epigenetic alterations within GA lesions cannot be ruled out and will warrant further investigation. In the meantime, we recommend that clinicians manage oral GA as a lesion with a low, if not null, risk of malignant transformation. Patient reassurance, together with elimination of potential risk factors (tobacco, alcohol, areca nut, and addressing GERD if diagnosed or suspected) are essential. Regular long-term follow-up is recommended. Excisional biopsy could be considered for small lesions, while larger lesions can be managed by “watchful waiting” following incisional biopsy.

A significant strength of this study is the relatively large sample size, which improves generalization of our findings and provides a robust basis for future research. Our work is also strengthened by rigorous clinicopathological correlation and the collaborative involvement of OMOP specialists, who submitted nearly all cases for histopathological analysis, ensuring high diagnostic accuracy. Another key strength is the strict case selection process in the literature review. Lesions consistent with other mucosal diseases were carefully excluded, allowing us to focus specifically on presumed cases of oral GA. This approach enhances the specificity and reliability of our conclusions.

Nonetheless, some limitations must be acknowledged. Many cases from the literature were excluded due to incomplete clinical or histologic documentation, absence of PAS and PAS-D staining, or likely misclassification, as some lesions initially reported as oral GA were more consistent with other well-defined white oral conditions (e.g., reactive keratoses, linea alba, leukoedema, or reactive/fibrous papules), including in the clinical context of Cowden syndrome. Additional exclusions included nodular or tumor-like clinical presentations, which are incompatible with the characteristic phenotype of GA. One further publication was excluded because it was written in Japanese, without sufficient translatable clinical or histologic detail to support accurate synthesis [9–12, 15–17, 21–23, 25, 26].

Among included cases, clinical photographs were inconsistently available [9, 13, 18, 20] requiring reliance on textual descriptions. Follow-up data was also incomplete, limiting our ability to assess long-term outcomes. Additionally, lifestyle factors such as tobacco and alcohol use were often not reported [9, 13, 20]. Some cases, especially those presented only as conference abstracts, were retrospective in nature and based on secondary data lacking comprehensive clinical details, with no subsequent full publications [9, 13, 18, 20]. Information on the presence or absence of GERD was inconsistently reported. The absence of reported GERD does not necessarily mean that a patient did not have GERD; therefore, we cannot reliably draw conclusions about a potential association between oral GA and GERD. These limitations collectively hinder the establishment of strong clinicopathologic correlations and preclude firm conclusions about long-term prognosis.

## Conclusion

Oral GA is likely a benign condition that should be considered in the differential diagnosis of a homogenous, milky-white, crisply-defined plaque in the oral cavity, especially on the lateral and ventral surface of the tongue. It often mimics OL and is therefore likely under-recognized by clinicians. However, the condition presents a distinctive histopathological profile characterized by parakeratosis, acanthosis and glycogen-rich squamous epithelial cells. Essential clinical and histopathological criteria are proposed for the first time to aid clinicians and pathologists in diagnosing this condition. We recommend that clinical management include patient reassurance, minimizing potential risk factors (tobacco, alcohol, GERD), limiting excisional surgeries and ensuring regular long-term follow-up.

Future research should prioritize prospectively collected, well-documented case series using a standardized diagnostic protocol, including histopathologic confirmation with PAS and diastase staining, such as the one we propose. Studies should apply uniform diagnostic criteria and incorporate molecular profiling to identify genetic or epigenetic alterations, clarify pathogenesis, and determine whether oral GA represents a purely reactive lesion or carries a predisposition to malignant transformation. This approach is essential to understanding the true behavior of this little-known entity and to establishing evidence-based guidelines for the diagnosis and management of oral GA. Endoscopic evaluations, including upper gastroscopy, may be warranted in selected patients, particularly those with GERD or suggestive symptoms, to assess for possible esophageal involvement. Further investigation is also needed to elucidate potential systemic associations and accurately determine the true prevalence of oral GA, especially in high-risk or immunocompromised populations.

## Supplementary Information

Below is the link to the electronic supplementary material.

Supplementary Material 1

## Data Availability

The data supporting the findings of this retrospective study consist of patient clinical records and histopathology slides that contain potentially identifiable information. Due to ethical restrictions and institutional privacy policies, these data cannot be made publicly available. De-identified data may be provided by the corresponding author upon reasonable request and with approval from the institutional ethics committee. The literature data supporting the analysis are available in the cited published studies.
