# Supplementary material for: Oral Glycogenic Acanthosis: A Clinicopathological Analysis of 13 Cases and Narrative Literature Review
Source: Head Neck Pathol. 2026 Feb 19;20(1):23. doi: 10.1007/s12105-026-01891-6 (PMC12921078; doi:10.1007/s12105-026-01891-6)
Supplement: Supplementary file 1 — Supplementary Material 1 [file 12105_2026_1891_MOESM1_ESM.docx]

Appendix A: Methodology of the narrative literature review

| **Database** | **Search Terms / Keywords** | **Period Covered** |
| --- | --- | --- |
| **PubMed (National Library of Medicine)** | (((oral) OR (extraesophageal)) OR ("Mouth"[Mesh])) AND (Glycogenic Acanthosis) | Inception – Sept 2025 |
| **Embase (via Ovid)** | (glycogen*.hw. and exp acanthosis/ ) OR ( (glycogen* adj2 acantho*).tw.) | Inception – Sept 2025 |
| **Google Scholar** | “oral glycogenic acanthosis” OR “glycogen accumulation oral mucosa” | Up to Sept 2025 |
| **Lens** | “extraesophageal glycogenic acanthosis” OR “glycogenic lesion oral cavity” | Up to Sept 2025 |
| **Manual Search (OOOO Journal; Head and Neck Pathology)** | Review of article titles and reference lists (2022–2025) | Jan 2022 – Sept 2025 |
